# Digital PCR: Endless Frontier of ‘Divide and Conquer’

**DOI:** 10.3390/mi8080231

**Published:** 2017-07-25

**Authors:** Peiyu Liao, Yanyi Huang

**Affiliations:** Beijing Advanced Innovation Center for Genomics (ICG), Biodynamic Optical Imaging Center (BIOPIC), College of Engineering, School of Life Sciences, and Peking-Tsinghua Center for Life Sciences, Peking University, Beijing 100871, China; peiyu@pku.edu.cn

**Keywords:** digital polymerase chain reaction (PCR), microfluidics, emulsion droplet, microwell chip

## Abstract

Digital polymerase chain reaction (PCR) is becoming ever more recognized amid the overwhelming revolution in DNA quantification, genomics, genetics, and diagnostics led by technologies such as next generation sequencing and studies at the single-cell level. The demand to quantify the amount of DNA and RNA has been driven to the molecular level and digital PCR, with its unprecedented quantification capability, is sure to shine in the coming era. Two decades ago, it emerged as a concept; yet one decade ago, integration with microfluidics invigorated this field. Today, many methods have come to public knowledge and applications surrounding digital PCR is mounting. However, to reach wider accessibility and better practicality, efforts are needed to tackle the remaining problems. This perspective looks back at several inspiring and influential digital PCR approaches in the past and tries to provide a futuristic picture of the trends of digital PCR technologies to come.

As the knowledge of molecular genetic scripts accumulates, the quantification of DNA and RNA molecules has become increasingly important. Numerous methods have been developed wittingly or not, to quantify the amount of nucleic acids as precisely as possible. Among the sequence-specific quantification methods, polymerase chain reaction (PCR)-based techniques have always been a favored option. Real-time quantitative PCR is regarded as a routine practice in biomedical laboratories. However, due to its limited counting resolution, this method does not meet the ever more stringent quantification demand, especially when the target concentration is relatively low or PCR inhibitors exist to perturb the exquisite assay. Moreover, as the techniques such as next generation sequencing and single-cell analyses continue to flourish, interest in quantification of nucleic acids has been drawn to the unprecedented single-molecule level. This has given rise to the prosperity of digital PCR technologies.

‘Limiting dilution PCR’ and ‘single-molecule PCR’ were the first names used to describe the method, until a much more popular and apposite term—‘digital PCR’—was put forward, which soon seized broad attention [1]. The strategy is simple, and the metaphor ‘divide and conquer’ is a good analogy [2]. DNA samples are separately amplified in independent yet identical partitions, and the all-or-none detection results of each reaction follow Poisson statistics. After counting the sum of positive reactions, by Poisson correction, not only the concentration but the absolute number of target molecules can be obtained. 

In early demonstrations [3,4], researchers diluted the samples into a large number (typically hundreds) of reaction vessels in micro-well plates (Figure 1a), the assay then was inevitably laborious and costly, and the partition number was thus limited. The situation changed when microfluidics were employed: reactor multiplication became division, and the total volume of the aqueous sample was therefore reduced to less than 100 µL.

## Digital PCR Met Microfluidics

Microfluidic-based digital PCR approaches generally fall into two categories, solid micro-chambers and water-in-oil emulsion. The former typically relies on microfabrication methods to create miniaturized micro-well devices with much smaller wells that are greater in number than our routinely used format (Figure 2). Such devices are commonly made of polydimethylsiloxane (PDMS), while glass and plastics have also been used. Aqueous samples are divided into portions of minute volume and partitioned in the chambers before they undergo PCR thermal cycling. Water-in-oil emulsion digital PCR comes after its counterpart in solid chambers but has developed rapidly since its advent. Flow focusing geometry is most frequently present in droplet generating microfluidic devices (Figure 2a). At the cross-shaped nozzle, the aqueous sample (the disperse phase) is pinched by oil (the continuous phase) and forms droplets. In this fashion, uniform droplets can be continuously generated at high speed under a stable driving force such as pumping pressure. For any methods to partition the water-based sample, volume and number of partitions, uniformity, and robustness are the most essential prerequisites for digital PCR. The reasons are that Poisson distribution has well determined that this method would innately favor smaller partitions in greater number for finer counting resolution and higher dynamic range, and that the thermal cycling procedure would incur considerations such as water evaporation, thermal stability of emulsion or microfluidic chips, and compatibility of thermal cycler, etc. These reasons defined the strategies brought up towards better digital PCR approaches.

**Figure 1:**
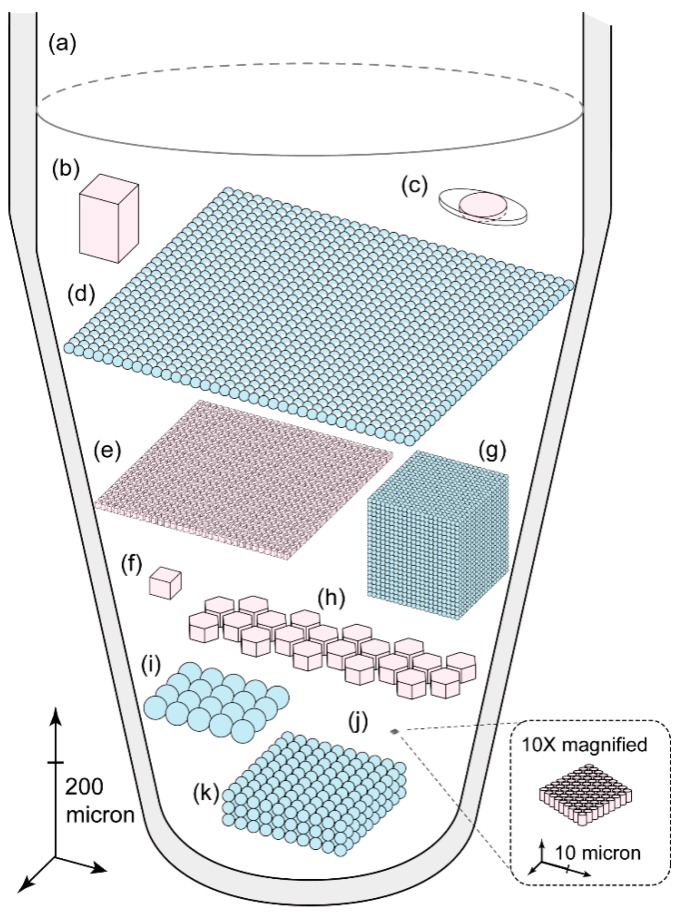
An overview of representative digital PCR approaches. For each partition shown herein represents approximately 1000 partitions in the corresponding methods. Their sizes too roughly reflect those in real-world situations. Micro-chambers in microfluidic chips are shown in pink and emulsion droplets in light blue. (**a**) Digital PCR in well plates [3,4]; (**b**) The first microfluidic chip for digital PCR in 2006 [5]; (**c**) SlipChip in 2010 [6]; (**d**) One-million droplet array in 2011 [7]; (**e**) Megapixel digital PCR in 2011 [8]; (**f**) Digital Array IFC (type: qdPCR 37k) from Fluidigm’s Biomark HD (Fluidigm Corporation, South San Francisco, CA, USA); (**g**) RainDrop digital PCR from Raindance Technologies (Lexington, MA, USA); (**h**) QuantStudio 3D from Life Technologies (now Thermo Fisher, Waltham, MA, USA); (**i**) QX200 droplet digital PCR platform from Bio-Rad (Hercules, CA, USA); (**j**) The densest and smallest microwell-based microfluidic digital PCR to date in 2012 [9]; (**k**) Chip-free digital PCR in droplets generated via micro-channel array (MiCA) centrifugation in 2017 [10].

Patterning micron-scale features is no hard job for the prevailing lithography techniques. A notable pioneering integration between digital PCR and microfluidics was reported in 2006 by Ottesen et al. (Figure 1b) [5]. They used a ’Quake valve’ structure that was made of multi-layer soft-lithography to actively separate individual reaction chambers. On one chip, 12 identical sections—each of about a thousand 6.5-nL partitions—can be processed simultaneously. This design was further modified in its commercial version. Slightly compromising the partition number per sample, Digital Array IFCs from Fluidigm’s Biomark HD made its chip more compact with more samples on one chip, whose most compact type is capable of 48 samples of 770 0.85-nL partitions (Figure 1f).

To enable digital PCR assay in a laboratory with insufficient expertise in microfluidics one may like to devise a method of sample partitioning free of pumps, neither hydraulic nor pneumatic. The SlipChip (Figure 1c) needs no exquisite microfluidic processes after chip fabrication, for its sample loading is just pipetting, and then a simple slipping [6]. SlipChips provide an obvious advantage for their readiness in resource-limited scenarios. Later, an idea called multivolume digital PCR [11,12] came to the researchers as how to minimize the size of the SlipChip and reduce the well number without compromising the performance specifications such as dynamic range. The key point is that the wells in the chip were designed to have different volumes in order to eliminate serial dilution. Simple as the authors claimed, it involves somewhat complicated mathematical derivation in theory and less intuitive interpretation of its results in practice.

The direct approach to wider dynamic range of digital PCR is to augment the amount of partitions. In 2011, the number was boosted to over a million as was described as “megapixel digital PCR” (Figure 1e) [8]. Valve-free partitioning on chip was realized by using fluorinated oil to flush the aqueous sample in the fluorophilic channels. The small volume of the chambers (10 pL) allowed the device’s high reactor density (4400 per mm^2^). PDMS’s permeability of gas and vapor allowed the ‘dead-end’ design—there were only inlets but no outlets so ambient air in the chambers was squeezed out through the silicon rubber—yet led to the addition of a parylene layer as a vapor barrier to prevent sample evaporation. 

Emulsion droplet’s capability to reach the same scale of partitioning was validated in the same year. Hatch et al. fabricated a microfluidic chip with 256 droplet generating nozzles that swiftly generated one-million 50-pL droplets (Figure 1d) and captured the fluorescence signals using wide-field imaging [7]. However, PCR that occurs in water-in-oil droplets was not unusual before its application in digital PCR. Emulsion PCR had been widely used in the next generation sequencing field to generate clonally amplified fragments [1]. Raindance Technologies was one of the earliest to use uniform sized water-in-oil droplets for digital PCR [13]. Years later in their commercialized emulsion generator, the droplet volume was reduced to 5 pL, and for a sample of about 70 µL, there were more than 10-million droplets (Figure 1g), which currently marks the record of the highest dynamic range of digital PCR platforms. 

In the pursuit of ever smaller partitioning and the extreme of miniaturization of the digital PCR devices, Men et al. fabricated the smallest wells (36 femtoliters) on a PDMS-made microfluidic chip with the densest well arrangement to date (over 20,000 reactors per mm^2^) (Figure 1j) [9]. Each device had over 80,000 hexagonally arrayed round-shaped wells of 3.3 µm in diameter and 4.2 µm in depth, using hydraulic pressing valve to actuate the compartmentalization and to prevent the evaporation through PDMS [9]. However, although pursuing limits in science is always fun, such a fine microfluidic device is by no means to be widely used, because for a popular method practicality and accessibility are the priorities. 

## Proper Design of Digital PCR

Although academia is good at competing over performance specifications, industry seems to know better what is ‘just good enough’ for a digital PCR solution. The latter, however, has to pay much more attention to the facility’s robustness, handiness, cost, and affordability to users, so trade-offs are inevitable. Two representative digital PCR platforms are QuantStudio 3D from Life Technologies (now Thermo Fisher) (Figure 1h) and Bio-Rad’s QX200 [14] (Figure 1i). The two coincide in partition volume (about 0.8 nL) and number (about 20,000) but adopt different strategies, the former emulsion droplet and the latter micro-well chip. It seems that their specifications of partition would be most welcomed by biologist users, whose favored sample volume is approximately 20 µL and concentration of interest would fall in this dynamic range in most cases. To the author’s knowledge, Bio-Rad may hold the biggest market share in the last two years and it is becoming popular in publications involving digital PCR.

Thanks to marketing propaganda, digital PCR has been well-known to many along with its competency in various applications. Still, apprehensions have kept many watching. Digital PCR seems to be in the similar case that quantitative PCR once was two decades ago, when few could afford to have a fancy machine on their own, but today this machine has become ubiquitous. Price hinders digital PCR’s popularity. In the past decade, cost per sample has dramatically decreased to 3 US dollars (Bio-Rad) [2], but the instrument price of the commercial platforms still remains much higher than quantitative PCR machines. To reduce the expenditure on microfluidics, Life Technologies set out to use silicon and Bio-Rad plastic for their partitioning units, respectively. Moreover, the field of microfluidics may not luckily follow Moore’s Law, and the cost may linger unless breakthroughs are to come.

Another factor that worries users is that, for emulsion based digital PCR, contamination seems likely to occur. Bio-Rad’s whole set contains two separate machines for droplet generation and reading. The droplets of different samples are first transferred by pipetting to a 96-well plate to be thermal cycled, and then droplets are sucked through one syringe nozzle and then serially interrogated at the fluorescent detecting spot. The exposure of amplified DNA products to open experimental environments risks contaminating subsequent assays. It is questionable whether such practice would be welcomed in clinical occasions. Besides, only part of the nucleic acid molecules of interest partake in the quantification assay. For example, in some micro-well partitioning approaches, excess sample was added to make sure all the chambers are taken, and much was removed in the sealing process. Likewise, in the emulsion droplet method involving microfluidic control, fluid waste exists before flow stabilizes to uniformly generate droplets, in addition to droplet breakage and loss amid emulsion transfer and mobile droplet reading. The effective readout rate is therefore limited to around 80% or less. This would not perturb Poisson statistics but is still a nuisance unexpected in stringent assays. 

## Turning Tides

Late-comers in the digital PCR industry have taken the lessons and made some adaptations. Clarity™ digital PCR (JN Medsys, Singapore) put forward a new method of micro-well partitioning: chip-in-a-tube [15]. A miniaturized chip with 10,000 micro-well that partitions samples but can be placed in the commonly used 200-µL PCR tube, where thermal cycling and signal reading take place. By this means, post-amplification DNA product is sealed throughout the process and normal thermal cyclers can be used. Crystal™ Digital PCR from Stilla Technologies (Villejuif, France) [16] gave another novel droplet generation method other than the conventional ones such as flow focusing, T-junction, or co-flow design. Gradient of confinement was the driving force and gave rise to uniform emulsion droplet generation due to insensitivity to the physical fluid properties [17]. In this method, droplet generation, thermal cycling, and reading happen in a planar flow cell chip in succession, prohibiting carryover contamination by closed processing. 

An innovation in monodisperse emulsion generation in 2016 told another story of droplet digital PCR by abandoning the ‘chip’ format. The device called MiCA has a small glass plate on which there are several through-holes of micron-level diameter [10]. Using centrifugal force to drive the process, aqueous sample is pinched off into monodisperse droplets and jets into surfactant-oil mixture beneath (Figure 1k) [10]. The aqueous sample could be almost completely transformed into droplets while the subsequent reading process rendered the readout rate less perfect, slightly higher than 80% (still higher than many other methods, though) [10]. This centrifugation droplet generation method manifested itself as a new possibility for it is a chip-free, pump-free, and loss-free protocol. Besides, multiple such MiCA tube can work simultaneously in a multiplex way, which is a good sign for high throughput, and the work has also validated that fluorine-free oil phase is compatible of digital PCR, which might help to reduce the oil cost.

In respect to the oil phase used in droplet digital PCR, fluorinated oil has dominated in the past decade. This inert and non-toxic liquid has appreciable performance in microfluidics and has been utilized since the early stage of digital PCR [13]. Its thermal stability, coalescence resistance, low viscosity and gas dissolving capacity contribute to its wide acceptance. Yet some factors keep fluorinated oil from being ideal. One is the high cost, which subject to the relatively tricky fluorine chemistry, would not see considerable decrease. Besides, compatible surfactant for fluorinated oil has to be fluorinated as well and hence is limited to few fluorinated proprietary products. The other, seemingly trivial issue is the high density. Aqueous droplets would gather to the surface of the emulsion and are prone to evaporate and shrink in size due to the large surface-to-volume ratio of emulsion droplets. Hydrocarbon-based emulsion oil has more surfactant options [7,18] and most of them are inexpensive and quite accessible. Also, droplets would not float near the surface but settle at the bottom of the low-density oil, resulting in less evaporation. Given this comparison between the two oil categories, it is still unforeseeable which would be the final winner for digital PCR emulsion, and there remains much room for improvement for the emulsion formulae.

**Figure 2:**
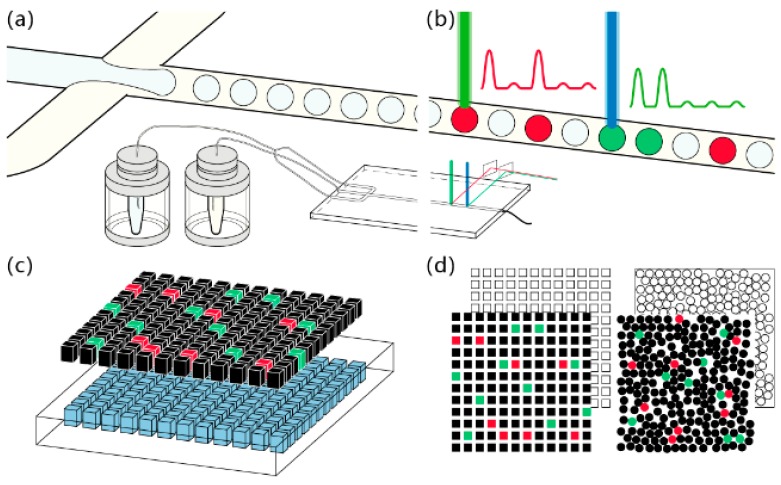
Microfluidic digital PCR partitioning and counting strategies. (**a**) Emulsion droplet generation using flow-focusing microfluidic chip; (**b**) Serial droplet fluorescence reading; (**c**) Minute volumes of PCR sample are partitioned in micro-well microfluidic chip; (**d**) Planar imaging for fluorescence positive chamber counting.

Digital reading or counting of the partitions so far can be generally categorized into two strategies: serial counting and planar imaging (Figure 2b,d, respectively). Regarding the reading scheme, few surprises were brought in the past decade, for these extant strategies seem to have effectively addressed this problem. Micro-well partitioning is a natural fit for planar imaging while its use of serial reading sounds less sensible. For droplet partitioning, most cases opted for serial counting. The aforementioned one-million-droplet digital PCR [7] had to use wide-field optics to image the single layer of close arrayed droplets. That is because if droplets are stacked during imaging, refraction and diffraction induced blurring would interfere with the imaging results and subsequent graphical analysis would be unreliable. There is a dilemma, that droplet digital PCR has a lower cost in partitioning but higher cost in reading, and micro-well digital PCR the other way around. This complex yet interesting problem will have to be overcome by the innovations to come. 

In summary, digital PCR has passed its emerging phase and is now trending profoundly, yet it is still not in its peak. Before meeting microfluidics, digital PCR was almost dormant. The past decade has seen how microfluidic techniques boosted biomedical development and digital PCR becomes an ideal example. Current methods have, to a great extent, lowered the cost per sample, which is mainly subject to the consumables. Digital PCR assay’s consumables are unlike common molecular biology assays, for they require delicate microfabrication, surface treatment, and special chemistry. To accomplish extraordinary performance specifications would sooner or later be less interesting since many boundaries have been explored. New trends will be to further lower the cost to greatly improve accessibility, and to make the hands-on process as fool-proof and contamination-proof as possible. These trends do not come from nowhere. For example, next generation sequencing has been driving the interest of quantity to a few-cell level, single-cell level, and even few-molecule level. Digital PCR’s applications are far reaching: its capability of absolute quantification, single nucleotide polymorphism genotyping, and copy number variation defines its significance in genomics, genetics, and diagnostics. As applications of digital PCR increase, so will the market.
